# Characterization and Genetic Diversity of *Listeria monocytogenes* Isolated from Cattle Abortions in Latvia, 2013–2018

**DOI:** 10.3390/vetsci8090195

**Published:** 2021-09-14

**Authors:** Žanete Šteingolde, Irēna Meistere, Jeļena Avsejenko, Juris Ķibilds, Ieva Bergšpica, Madara Streikiša, Silva Gradovska, Laura Alksne, Sophie Roussel, Margarita Terentjeva, Aivars Bērziņš

**Affiliations:** 1 Institute of Food Safety, Animal Health and Environment BIOR, LV-1076 Riga, Latvia; jelena.avsejenko@bior.lv (J.A.); juris.kibilds@bior.lv (J.Ķ.); ieva.bergspica@bior.lv (I.B.); madara.streikisa@bior.lv (M.S.); silva.gradovska@bior.lv (S.G.); laura.alksne@bior.lv (L.A.); aivars.berzins@bior.lv (A.B.); 2 Institute of Food and Environmental Hygiene, Faculty of Veterinary Medicine, Latvia University of Life Sciences and Technologies, LV-3004 Jelgava, Latvia; Margarita.Terentjeva@llu.lv; 3 Maisons-Alfort Laboratory of Food Safety, University Paris-Est, French Agency for Food, Environmental and Occupational Health (ANSES), F-94701 Maisons-Alfort, France; sophie.roussel@anses.fr

**Keywords:** *Listeria monocytogenes*, cattle, abortion, genetic diversity, seasonality

## Abstract

*Listeria monocytogenes* can cause disease in humans and in a wide range of animal species, especially in farm ruminants. The aim of the study was to determine the prevalence and genetic diversity of *L. monocytogenes* related to 1185 cattle abortion cases in Latvia during 2013–2018. The prevalence of *L. monocytogenes* among cattle abortions was 16.1% (191/1185). The seasonality of *L. monocytogenes* abortions was observed with significantly higher occurrence (*p* < 0.01) in spring (March–May). In 61.0% of the cases, the affected cattle were under four years of age. *L. monocytogenes* abortions were observed during the third (64.6%) and second (33.3%) trimesters of gestation. Overall, 27 different sequence types (ST) were detected, and four of them, ST29 (clonal complex, CC29), ST37 (CC37), ST451 (CC11) and ST7 (CC7), covered more than half of the *L. monocytogenes* isolates. Key virulence factors like the *prfA*-dependent virulence cluster and *inlA, inlB* were observed in all the analyzed isolates, but *lntA, inlF, inlJ, vip* were associated with individual sequence types. Our results confirmed that *L. monocytogenes* is the most important causative agent of cattle abortions in Latvia and more than 20 different STs were observed in *L. monocytogenes* abortions in cattle.

## 1. Introduction

*Listeria monocytogenes* is a gram-positive, facultative intracellular pathogen that is ubiquitously distributed in the environment. The natural habitat of the pathogen is thought to be decomposing plant material in which *L. monocytogenes* exists as a saprophyte [1]. It is well-known as a zoonotic pathogen as it can cause listeriosis—an infectious disease of humans and different animal species [2,3,4,5]. Listeriosis in animals can be observed in various clinical manifestations—uterine infection, septicemia, central nervous system infection, gastroenteritis, mastitis and eye infection [6,7,8,9]. A uterine infection can result in stillbirth, septicemia in neonates and abortion, most often in the last trimester of gestation [10]. Farm ruminants, especially cattle, sheep and goats, are the most frequently affected [3]. Furthermore, clinically healthy farm ruminants can shed *L. monocytogenes* in the environment with feces and therefore are considered to be a natural reservoir and vector of *L. monocytogenes* at farms [4,11,12,13]. The pathogen is ingested *via* the oral route and the contaminated silage is assumed to be one of the main sources of cattle listeriosis at farms [14].

The ability of *L. monocytogenes* to tolerate different environmental conditions like oxygen levels, temperature and humidity makes it difficult to eliminate the pathogen at farms and food processing facilities. Noteworthily, economic losses in livestock occur due to *L. monocytogenes*-caused morbidity and mortality [5,15]. *L. monocytogenes* excreted by livestock can contaminate milk and thus subsequently the pathogen can enter the food production chain and pose a potential health risk to humans [15].

The clonal structure of *L. monocytogenes* is extensively studied. Multilocus sequence typing (MLST) organizes strains into clonal complexes (CCs) and it has been shown that several clones are associated with the neurological (CC1 and CC6) and maternal–neonatal forms of listeriosis (CC1, CC2, CC4) and food (CC121, CC9) [16]. MLST and other genotyping methods have also allowed reconstruction of the evolution of *L. monocytogenes* where most strains can clearly be assigned to one of four genetic lineages. Isolates belonging to lineage I are observed in both human and animal infections and are reported to be present at dairy farms. Lineage II isolates are frequently isolated from the environment, animal infections and food and are also linked to human listeriosis cases. Strains belonging to lineages III and IV are much less common [17,18,19,20].

New typing methods are developing fast and whole genome sequencing (WGS) is replacing other methods like serotyping and pulsed field gel electrophoresis (PFGE) worldwide. WGS analysis offers the highest discriminatory power among different organisms and is successfully applied in foodborne outbreak investigations and molecular epidemiology of pathogenic bacteria including *L. monocytogenes* [21,22,23]. The Institute of Food Safety, Animal Health and Environment BIOR is the national reference laboratory for *L. monocytogenes* in food and animals and implements state surveillance, including the animal diseases surveillance program. Implementation of routine WGS provides a lot of data to better understand the processes of pathogen evolution and distribution. It is important to analyze retrospective pathogen collection as well as prospective samples and assess the effectiveness of the restrictions applied to eliminate them.

The aim of the study was to assess the prevalence and genetic diversity of *L. monocytogenes* related to abortions in cattle by means of WGS analysis. This is the first study in Latvia investigating *L. monocytogenes* isolated from animals and the results provide valuable information for further research in this field.

## 2. Materials and Methods

### 2.1. Sampling

*L. monocytogenes* isolates were collected within the frame of the state surveillance program of the Republic of Latvia for the investigation of cattle abortion cases within a six years’ period (2013–2018). The state surveillance program provided that cattle owners should notify a veterinarian about all the observed abortion cases, then an official veterinarian would collect the aborted fetus or placenta (in case the fetus was not available) and transport samples to the laboratory. The state surveillance program covered microbiological investigation of aborted fetuses; therefore, the necropsy was carried out only for the purpose of obtaining samples for microbiological analyses. The definition of *L. monocytogenes*-caused abortion was based on laboratory diagnosis—isolation of *L. monocytogenes* from tissues of the aborted fetus or placenta if the fetus was not available. A total of 1185 cattle abortion cases were investigated during the studied period. The data about the animals’ age, gestation period and season of abortion were collected for the surveillance purposes.

The years were divided into seasons according to the Latvian Environment, Geology and Meteorology Centre data: winter (December–February), spring (March–May), summer (June–August) and autumn (September–November).

### 2.2. Microbilogical Analyses

Isolation of *L. monocytogenes* was performed according to the methodology described in ISO 11290-1 (2017) and the OIE Manual of Diagnostic Tests and Vaccines for Terrestrial Animals (Chapter 3.10.5, 2021). The necropsy material from an aborted fetus included heart, liver, spleen, kidney and lung samples and was used for microbiological analyses. For the bacteria isolation procedure, 10–25 g of pooled samples of the fetus tissue or placenta were aseptically transferred into sterile stomacher closure bags, half Fraser broth was added for enrichment to achieve a 1:10 dilution, and then they were homogenized by stomaching (BagMixer^®^ 400, Interscience, Saint-Nom-la-Breteche, France) at normal speed for 60 sec. After incubation at 30 °C for 24 h, 0.1 mL of enrichments (Biolife, Milan, Italy) in half Fraser broth were transferred to 10 mL of Fraser broth and incubated at 37 °C for 24 h. After incubation, 0.01 mL (using a 10 µL loop) of enrichments from Fraser broth were plated onto two selective *Listeria* agars according to the Ottaviani and Agosti (ALOA) and OXFORD (Biolife, Milan, Italy) formulations and incubated at 37 °C for 24–48 h. Small and round (0.5–1 mm in diameter) colonies of blue–green color surrounded by an opaque halo on the ALOA medium and olive-colored colonies on the OXFORD medium were considered as *Listeria* spp. and plated onto sheep blood agar. The colonies which were small, round (0.5–1 mm in diameter), of grey or grayish white color, showing hemolysis on the sheep blood agar medium were considered to be *Listeria* spp. Presumptive colonies of *Listeria* spp. were confirmed and identified with a matrix-assisted laser desorption/ionization time-of-flight mass spectrometry (MALDI TOF MS) Biotyper (Bruker, Bremen, Germany).

### 2.3. Whole Genome Sequencing and Genomic Analysis

From the total of 191 isolated *L. monocytogenes* strains in cattle abortions, 157 were available in the biobank and complemented by metadata and therefore selected for sequencing. The obtained sequencing data were subjected to quality control (criteria listed below) and the sequences which did not match the quality criteria or belonging to other *Listeria* species were excluded from the dataset. Other *Listeria* species were identified in eight of the 157 sequences (5.1%)—two *L. innocua* and six *L. seeligeri* sequences. In total, 125 *L. monocytogenes* isolates were used for further analysis in our study. In the years 2013–2015, multiplex PCR [24] was used for serogroup differentiation of *L. monocytogenes*; however, these results were compliant with in silico serotyping of genomes; therefore these results are not presented separately.

DNA for WGS was extracted from single colonies grown overnight on sheep blood agar at 37 °C. A QIAamp DNA Mini Kit (Qiagen, Hilden, Germany) was used according to the manufacturer’s protocol for gram-positive bacteria. Genomic libraries were prepared using Nextera XT chemistry (Illumina, San Diego, CA, USA) and sequenced on an Illumina MiSeq using a 600 cycle V3 kit to obtain 2 × 300 bp paired-end reads. On average, 100,000 reads were generated for each bacterial isolate, resulting in the median genome coverage of 50×. Low-quality bases and adapters were removed from raw reads using Trimmomatic v0.38 [25]. The trimmed reads were *de novo* assembled into contigs by the SPAdes assembler (v3.14.0) [26]. Multiple quality control steps were performed to exclude contaminated or wrongly identified genomes from downstream analysis. Namely, the total assembly size and N50 (required 10 kb or above) were checked with QUAST v5.0.1 [27], and contamination and species identification were checked by comparing the raw reads against the MiniKraken (v1_8GB_201904) taxonomic database with Kraken v2.0.8 [28]. The genome assemblies that passed all the quality requirements were imported into the SeqSphere+ 7.0.4 software (Ridom, Germany) [29] for serotype prediction, seven-gene multilocus sequence typing (ST), clonal complex (CC) and core genome MLST (cgMLST) typing; cgMLST alleles were identified according to the cgMLST scheme by Ruppitsch et al. (2015) [30]. Core genome allele diversity and relationships between the isolates were visualized using a minimum spanning tree. The tree layout was constructed using the MSTree V2 algorithm implemented in Grapetree [31].

### 2.4. Virulence Factor Analysis

Presence of virulence factors was determined by querying translated genomic DNA sequences against protein sequences of known *Listeria* virulence factors using the DIAMOND software (version 2.0.9) [32]. Thresholds for minimum sequence identity and minimum target coverage were set to 90% and low-complexity sequence masking was disabled. Forty-two virulence factor amino acid sequences that were used in the DIAMOND search were retrieved from the Virulence Factor Database [33].

### 2.5. Statistical Analyses

Fisher’s exact test was performed to detect the significance of the association of *L. monocytogenes* abortions and the season.

## 3. Results

### 3.1. Prevalence of L. monocytogenes-Associated Cattle Abortions

**Figure 1:**
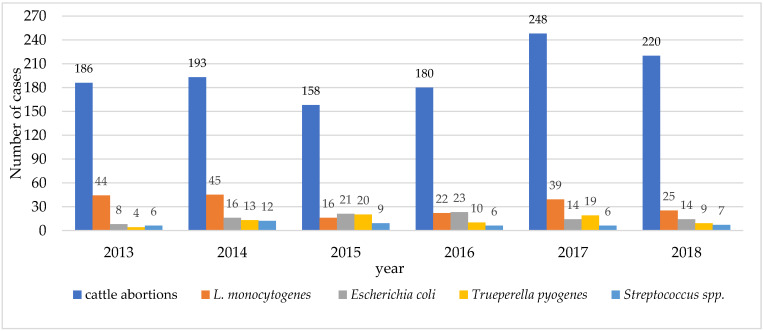
Investigated cattle abortion cases and the most frequently observed pathogens associated with cattle abortions (2013–2018).

From 150 up to 250 cattle abortions were reported per year from 2013 to 2018, with *L. monocytogenes* identified in 10.1–23.7% in years 2015 and 2017, respectively. The overall prevalence of *L. monocytogenes* was 16.1% (191/1185) (Figure 1) and it was the most frequently observed pathogen among cattle abortions. *Escherichia coli* and *Trueperella pyogenes* were identified in 8.1% (96/1185) and 6.3% (75/1185) of the cases, respectively. *Streptococcus* spp. was observed in 3.9% (46/1185) of all the cattle abortions and corresponds to the fourth most commonly observed pathogen in cattle abortion cases.

Available metadata (Table S1) on cattle abortions to characterize listeriosis in ruminants are provided in the Supplementary Data.

Seasonality among *L. monocytogenes* cattle abortion cases was observed. *L. monocytogenes* were detected in 22.3% (84/376) of the cattle abortions during the springtime and comprised 7.1% (84/1185) of all the studied cases, whereas the prevalence of *L. monocytogenes*-caused abortion was 13.0% (45/347) in winter, 13.0% (30/231) in summer and 13.9% (32/231) in autumn. The prevalence of *L. monocytogenes*-caused abortions was higher in spring (*p* < 0.01) while differences in the prevalence of *L. monocytogenes*-caused abortions during the other seasons were not identified (*p* > 0.05).

The age of 118 affected cattle was known. Analyzing this dataset, *L. monocytogenes*-caused abortions were more frequently observed in the cattle under four years of age (61.0%, 72/118) than in the cattle five years and older (39.0%, 46/118).

The gestation period in which the abortion occurred was known in 48 cattle. The majority of *L. monocytogenes*-caused abortions was reported in the third trimester (31/48, 64.6%), followed by the second (16/48, 33.3%) and the first trimesters (1/48, 2.1%) of the cattle gestation period.

*L. monocytogenes* collection was obtained from 89 farms. At 75 farms, a single *L. monocytogenes*-associated abortion was observed and eight farms were with two *L. monocytogenes*-related abortion cases. However, at six farms (later in the text designated as A–F, Table S1 in the Supplementary Data), three or more isolates were obtained: 14 isolates at farm A, six isolates at farm B, four isolates at farms C and D each, and three isolates at farms E and F each.

### 3.2. Genetic Characterization of L. monocytogenes Isolates

Genetic diversity of cattle abortion *L. monocytogenes* isolates, including serogroup, ST (CC) and cgMLST, was analyzed based on the WGS data. *L. monocytogenes* isolates belonging to three different serogroups were identified. The majority of the isolates belonged to serogroup IIa (93.6%), followed by IIc (4.0%) and IVb (2.4%). Serogroup IIb was not identified in any of the *L. monocytogenes* isolates. All the isolates were found to belong to lineage II except for three isolates from the IVb serogroup that represented lineage I.

All the isolates were classified into 27 STs of 23 different CCs listed in Table 1. The most common STs were ST29 (CC29) and ST37 (CC37) each represented by 18 isolates, followed by ST451 (CC11) with 16 isolates and ST7 (CC7) with 13 isolates. The same STs were dominating at the farm level; each of them was observed at least at 15–17 different farms (Table S1 in the Supplementary Data). Most isolates from these four STs were ubiquitously spread all over the analyzed period and altogether they represented more than 50% of the analyzed isolates. Most of the other STs observed in cattle abortion cases were represented by 1–7 isolates in total of the analyzed period, up to two cases per year; however, in some cases, increases in individual STs were observed, for example, in ST689 (CC689)—seven cases in 2017, ST9 (CC9)—five cases in 2017, ST451 (CC11)—seven cases in 2017 (Table 1).

**Figure 2:**
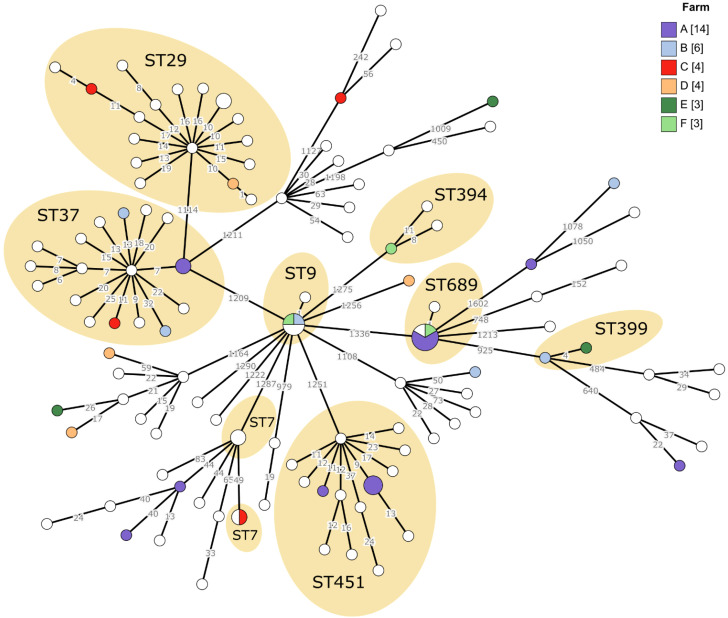
A minimum spanning tree showing the core genome allele diversity of *L. monocytogenes* isolates from cattle abortions. The tree is based on 1701 cgMLST loci. Farms with more than three isolates (designated as A–F) are represented with a color code, and the nod size is proportional to the isolate count per genotype. Depicted branch lengths are log-transformed but numbers on the branches represent the absolute distance between genotypes in the number of loci. The clusters within STs where the distance between a least two isolates is 10 alleles or less are highlighted as possible infection outbreak or transmission events.

Analysis of *L. monocytogenes* genomes at the cgMLST level revealed multiple clusters with between-isolate distances of 10 alleles or less, which is our standard cutoff for the cgMLST-based epidemiological analysis (Figure 2). A few clusters of identical genotypes among several farms were apparent, namely the clusters of ST9 and ST689 and two small clusters of ST7. The clusters made of ST689 and ST9 included seven and five isolates, respectively. Prevalence of these two STs increased substantially in 2017 (see Table 1), thus indicating a possible listeriosis outbreak. However, an epidemiological link for the cluster of ST689 was not supported by geographic location of the involved farms and for the ST9 cluster it was only partially supported by farm locations. The ST7 cluster consisting of two identical isolates and involving farm C is supported by infection timing but spatial connection seems not likely given that both farms are not located in adjacent regions. The other ST7 cluster, however, includes only two isolates from the same farm in the same year so a small-scale outbreak is likely to have happened. The cluster of ST399 includes two isolates with high genetic similarity from subsequent years but they were isolated in very distant regions. The ST394 cluster consists of three isolates from distant regions and different years but still they show high genotype similarities. Isolates from the ST451 cluster involving farm A originated from adjacent regions in subsequent years—transmission seems likely only if it was mediated via some persistent environmental reservoir or carrier. The group of ST29 isolates including one from farm D do not appear epidemiologically connected except for two which come from the same farm in the same year. Finally, the ST37 cluster seems like mostly sporadic cases due to being distributed over temporal and spatial scales.

Among the most prevalent STs (ST7, ST29, ST37 and ST451, each with more than ten isolates), an interesting pattern can be observed: the average minimum spanning tree branch length is 11–14 among the ST29, ST37 and ST451 isolates. However, the average branch length among the ST7 isolates is 34 alleles, indicating a more diverse clade and less likely epidemiological links within the ST7 group (a detailed view of the ST genotype of each isolate in the minimum spanning tree can be found in the Supplementary Data, Figure S1).

As can be seen in the minimum spanning tree of *L. monocytogenes* cgMLST, most strains originating from one farm were not closely related to each other, they often belonged to various STs even when isolated in one year (Figure 2, Farms B, C, D, E, F). The exception is farm A with 14 isolates within a six years’ period. Nine of these isolates were included in three different clusters (Figure 2, clusters of ST37, ST451 and ST689).

In total, 42 virulence factors (VF) were analyzed (Table S2 in the Supplementary Data). The key virulence factors like *prfA, hly, plcA, inlA, inlB* were observed in all the analyzed isolates independently of ST. Several genes were identified as ST-dependent, e.g., *lntA* was missing in all the isolates of ST18, ST21, ST207, ST391 and ST451 (summarized in Table 1). Internalin genes *intA*, *intB*, *intC, intK* and *intP* were detected in every analyzed ST and only genes *inlJ* and *inlF* presented the ST-dependent profile—both were missing in ST2, ST6, ST121, ST399 and ST689, while only *inlF* was missing in ST4, ST11, ST14. The single ST6 strain contained genes encoding listeriolysin S—*llsA, llsB, llsD, llsG, llsH, llsY* and *llsX*, while in ST4, only *llsD* and *llsG* were detected. Both ST6 and ST4 belong to serogroup IVb and are assumed to be more virulent than others; in other STs or individual isolates, LIPI-3 genes were not detected.

## 4. Discussion

Our study revealed that *L. monocytogenes* was the most prevalent pathogen in cattle abortions in Latvia. *L. monocytogenes*-caused abortions in ruminants have been reported worldwide and the occurrence rate has varied from sporadic cases to outbreaks [6,20,34]. *Escherichia coli, Trueperella pyogenes* and *Streptococcus* spp. were found among the four most frequently associated cattle abortion causative agents and this observation was similar to the study of Wolf-Jackel et al. [35] where *T. pyogenes* and *E. coli* also were among the most important causative agents of cattle abortions. In our study, the decreasing trends in occurrence of *L. monocytogenes*-caused abortions in cattle were observed through the years. Most often, cattle abortions due to *L. monocytogenes* were detected in 2013 and 2014 with the prevalence of 23.7% and 23.3%, respectively. In the following years, the pathogen was detected noticeably less often in cattle abortions—10.1% in 2015, 12.2% in 2016, 15.7% in 2017 and 11.4% in 2018. This observation could be explained by the fact that increased awareness and revision of biosafety measures were implemented to react to the ongoing worsening of the situation with African swine fever in Latvia that could be the reason for the improvement of hygienic measures at farms. Hygienic measures were reported to be an important tool for the reduction of spread of pathogens at farms.

The results of this study demonstrated the evident seasonality of *L. monocytogenes* occurrence among cattle abortions. We observed increased prevalence of *L. monocytogenes* abortions in spring (22.3%) compared to other seasons. The seasonal character of listeriosis clinical cases in winter and spring was reported previously in 44.2% and 38.4% of the cases, respectively [6], as well as the overall increase in *L. monocytogenes* prevalence in farm animals during the cold weather season [19,36,37,38]. These authors also highlighted feeding of silage and indoor keeping as the main factors which could promote the listeriosis onset during winter and spring. Any changes in the daily routine of herds, either in management, climate or physiological changes, can be a stress factor, affect the immune defense mechanism and predispose animals to infection with *L. monocytogenes* [3].

The gestation period of the aborted cattle was known in only 48 cases, but these data revealed a tendency that *L. monocytogenes*-caused abortions occurred during the second (33.3%) and the third (64.6%) trimesters of gestation. This observation was in agreement with previous studies which showed that *L. monocytogenes* abortions were more prevalent at the late term of gestation, mainly during the last trimester [10,34,39]. Abortions at the early stage of gestation could occur as an absorption of the fetus and fetal membranes; it should be taken into account that not all early-term abortions were detected and reported.

Despite the fact that the genetic diversity of *L. monocytogenes* isolates was rather high, ST29 (CC29), ST7 (CC7), ST37 (CC37) and ST451 (CC11) were continuously identified through each year of the study. Kim et al. (2018) carried out a 12-year study about genetic diversity of *L. monocytogenes* in bulk tank milk, milk filters and milking equipment from dairies in the United States and reported CC7, CC37, CC29 and CC451 as the four most frequently detected CCs in their study; CC7 and CC37 were also observed in each of the studied years [40]. Almost all of the observed *L. monocytogenes* strains in cattle abortion cases in our study were also reported by Painset et al. (2019) in the survey of *L. monocytogenes* in ready-to-eat foods and human clinical cases in Europe. In addition, CC7, CC14, CC415 and CC4 were associated with outbreaks in humans [22]. Papic et al. (2019) analyzed *L. monocytogenes* isolates of animal clinical cases and environment from France, Slovenia, Switzerland and Great Britain and found CC1 of serogroup IVb to be the most prevalent in both clinical forms of listeriosis—rhombencephalitis (57.6% of all the cases) and abortion (18.8% of all the cases) [20]. In another study, Dreyer et al. (2016) reported CC1 to be predominant in ruminant rhombencephalitis cases [18]. Serogroup IVb was found to be more prevalent than IIa among wildlife animal samples in Spain [41]. Hypervirulent clones of IVb were present in food and food production environments, highlighting their importance associated with human invasive listeriosis cases [42]. In our dataset, CC1 in the context of abortion was not observed at all, and neurological manifestations of listeriosis in cattle in Latvia are rarely reported, only one case since 2013, and in that case, we identified *L. monocytogenes* ST1 (CC1) (unpublished data, BIOR). Papic et al. (2019) analyzed *L. monocytogenes* isolates from several European countries and reported CC37 as the second most frequently observed in cattle abortions (15.6%) followed by CC4, CC217, CC6 and CC7. Therefore, CC37 and CC6 showed significant correlation with abortions in ruminants. Isolates of lineage I were the most prevalent among cattle abortions in European countries, followed by lineage II [20]. In comparison, abortions in beef heifers in the USA were associated with *L. monocytogenes* strains of lineages III and I [34]. In our study, isolates belonging to lineage II were predominant in cattle abortion cases and *L. monocytogenes* isolates of lineage I were observed sporadically. This finding is in line with the previously reported data [18,43] that lineage II is common in non-encephalitic infections, including ruminant abortions.

The diversity of strains in abortion cases suggests that there was a broad genetic diversity of *L. monocytogenes* in the farm environment [36,37]. In a study involving several European countries, the comparison of environmental *L. monocytogenes* strains and ruminant clinical strains revealed coincidence with the CCs observed in abortions, but the CCs related to rhombencephalitis were different from the environmental repertoire [20]. According to a study of environmental *Listeria* spp. isolates at Latvian cattle farms, the predominant *L. monocytogenes* strains were ST37 (CC37), ST451 (CC11) and ST18 (CC18) [44], which all were among the most prevalent STs in cattle abortions. Significantly, the STs mentioned above were present in all kinds of the investigated environmental samples. Nevertheless, ST7 (CC7) which was observed in 10.4% of the cattle abortions, was found to be present only in soil samples, but ST29 (CC29) with a constantly high prevalence in cattle abortions over years was detected in animal feces and water samples. Some of *L. monocytogenes* strains like ST1085 (CC1985), ST1482 (CC1482) and ST194 (CC315) were found to be present only in the environmental samples, but were not observed in the cattle abortion cases. Furthermore, Terentjeva et al. showed that lack of cleaning and disinfection of cattle feeding tables was associated with higher *L. monocytogenes* prevalence in soil samples of farms. The detection of the same STs in the environmental and clinical *L. monocytogenes* isolates highlights the importance of implementing and using appropriate hygiene measures to reduce contamination of the environment and prevent animal infection with *L. monocytogenes* [44].

Large genetic variety of *L. monocytogenes* was observed at the farm level; cgMLST analysis demonstrated most isolates representing individual cgMLST types that are consistent with the surveillance data—from 75 farms, one isolate per farm was analyzed. In cases where three or more isolates per farm were analyzed, they mostly represented unrelated *L. monocytogenes* clones. At farm A (Figure 2) with the highest *L. monocytogenes* abortion prevalence among the farms, five isolates were separated from each other but nine were included in three separate clusters. This is in line with the previous study of Castro et al., 2018, who reported different genotypes of *L. monocytogenes* per farm detected by PFGE [37]. It should be mentioned that farm A is a training and research farm for veterinary medicine students, therefore, higher prevalence of the infection could be explained by frequent changes in the personnel and possible bypass of the biosafety measures.

In our study, cgMLST clusters were investigated in order to discover patterns and potential instances of within-farm or between-farm transmission. After considering time and location of isolation in addition to the genotype data, most of these clusters do not seem to represent direct transmission events or outbreaks. However, striking genotype similarities were observed between some farms (Figure 2, ST9 and ST689 clusters). According to the study design, no information about the movement and distribution of animals or materials (e.g., feed) between the farms was obtained. However, it is possible that such information could provide additional insights on multi-farm clusters of identical or highly similar genotypes. An additional factor complicating cluster analysis is the possibility of asymptomatic carrier animals or unobserved first-trimester abortions which might link together cases that otherwise seem too far apart in terms of time or allelic difference. Similarly, environmental sources could also play an important role in the spread of *L. monocytogenes* as supported by the overlap between the most frequent STs of this study and isolates from the farm environment [44]. It remains to be clarified whether these STs are predominant among cattle abortion cases because they are widely distributed in environmental reservoirs or because they are more infectious and/or cause more severe symptoms and are thus easily detected by surveillance that targets clinical manifestations of listeriosis.

The virulence potential of *L. monocytogenes* strains varies. Some strains are naturally virulent and cause high morbidity and mortality rates, but other strains are non-virulent and unable to cause infection in mammals [45,46]. The main virulence factors like *prfA, hly, plcA, inlA, inlB* involved in the pathogenesis of *L. monocytogenes* were observed in all the analyzed isolates. *PrfA* is a major transcriptional activator of *L. monocytogenes* virulence genes [47,48]. When *L. monocytogenes* enters a host cell, it is primarily located in single-membrane vacuoles. Two virulence factors, listeriolysin O (encoded by *hlyA*) and phosphatidylinositol-specific phospholipase C (encoded by *plcA*), are important for lysis of the primary single-membraned vacuoles and subsequent escape of *L. monocytogenes*. After the lysis of primary vacuoles, *L. monocytogenes* is released to the cytosol, where intracellular growth and multiplication take place. Surface protein actin (actA) is implicated in the intracellular mobility and cell-to-cell spread of *L. monocytogenes* [2,49]. Internalins are a highly diverse gene family consistent with the phylogenetic lineages of *L. monocytogenes* and a previous study showed the *inlF* gene absent from lineage I isolates [50]. A secreted *Listeria* virulence factor encoded by the *lntA* gene is involved in modulating the host’s INF-λ-mediated immune response [51]. Virulence factor *vip* interacting with host cell endoplasmic reticulum resident chaperone Gp96 is involved in cell invasion and/or signaling events that may shape the host’s immune response during infection [52]. Listeriolysin S encoding gene family *lls* is responsible for the interaction of bacteria with gut microbiota—a significant factor in listeriosis pathogenesis [53]. Additionally, *vip* and *intF* were previously reported as significantly associated with human clinical and nonclinical cases, respectively [22]. Here, we reported ST-dependent presence/absence of virulence genes in *L. monocytogenes* genomes that can contribute to infectivity and severity of infection; however, additional genome and patho-clinical data analyses are necessary to detect specific gene variants and mutations that can contribute to the pathogenesis of *L. monocytogenes*.

## 5. Conclusions

Our study confirmed that *L. monocytogenes* is the most important causative agent of cattle abortions in Latvia with the significantly highest (*p* < 0.01) occurrence in spring. A broad genetic variety between isolates was observed between farms and within farms. In this study, the most prevalent STs, ST37, ST29, ST451, ST7, were previously reported in the association with the environment and dairy production, but a few hypervirulent strains such as ST6 were detected as well. The main virulence factors involved in the pathogenicity mechanisms of *L. monocytogenes* were present in all the isolates, but several virulence factors were determined as ST-specific traits. In order to strengthen the One Health approach and promote disease prevention in animals as well as in humans, the measures to diminish *L. monocytogenes* infection should be considered already at the farm level, reducing the possibility of the pathogen to enter the food chain.

## Figures and Tables

**Table 1:** *L. monocytogenes* isolates obtained from cattle abortion cases classified by the serogroup, clonal complex (CC), sequence type (ST), lineage and distribution of virulence factors (VF), *n* = 125, in Latvia, 2013–2018.

Serogroup	CC	ST	Lineage	VF	2013	2014	2015	2016	2017	2018	Total
IIa (*n* = 117)	CC7	ST7	II	lntA+, vip–, inlJ+, inlF+	2	4	1	2	1	3	13
	CC8	ST8	II	lntA+, vip–, inlJ+, inlF+	2	2	1	2	-	-	7
		ST120	II	lntA+, vip–, inlJ+, inlF+	-	1	-	-	-	-	1
	CC11	ST11	II	lntA+, vip+, inlJ+, inlF–	-	1	-	1	1	-	3
		ST451	II	lntA–, vip+, inlJ+, inlF+	1	2	1	2	7	3	16
	CC14	ST14	II	lntA+, vip+, inlJ+, inlF–	-	-	-	1	1	1	3
		ST91	II	lntA+, vip+, inlJ+, inlF+	-	2	-	1	-	-	3
		ST399	II	lntA+, vip+, inlJ–, inlF–	-	-	-	1	1	-	2
	CC18	ST18	II	lntA–, vip+, inlJ+, inlF+	2	-	2	-	1	1	6
	CC19	ST19	II	lntA+, vip–, inlJ+, inlF+	-	-	1	-	-	-	1
	CC20	ST20	II	lntA+, vip+, inlJ+, inlF+	1	2	-	2	1	-	6
	CC21	ST21	II	lntA–, vip–, inlJ+, inlF+	-	1	-	-	1	-	2
	CC29	ST29	II	lntA+, vip–, inlJ+, inlF+	-	5	1	4	4	4	18
	CC37	ST37	II	lntA+, vip–, inlJ+, inlF+	3	4	1	2	3	5	18
	CC121	ST121	II	lntA+, vip+, inlJ–, inlF–	-	2	-	-	-	-	2
	CC207	ST207	II	lntA–, vip+, inlJ+, inlF+	-	1	-	-	-	-	1
	ST226	ST226	II	lntA+, vip–, inlJ+, inlF+	-	1	-	-	-	-	1
	CC89	ST391	II	lntA–, vip–, inlJ+, inlF+	-	-	-	1	-	-	1
	CC415	ST394	II	lntA–, vip+, inlJ+, inlF+	1	-	-	-	2	-	3
	CC403	ST403	II	lntA+, vip–, inlJ+, inlF+	-	-	-	1	-	-	1
	CC90	ST425	II	lntA+, vip+, inlJ+, inlF+	-	-	-	-	1	-	1
	CC573	ST573	II	lntA+, vip–, inlJ+, inlF+	-	-	-	1	-	-	1
	CC689	ST689	II	lntA+, vip+, inlJ–, inlF–	-	-	-	-	7	-	7
IIc (*n* = 5)	CC9	ST9	II	lntA+, vip+, inlJ+, inlF+	-	-	-	-	5	-	5
IVb (*n* = 3)	CC2	ST2	I	lntA+, vip–, inlJ–, inlF–	-	-	1	-	-	-	1
	CC4	ST4	I	lntA+, vip–, inlJ+, inlF–, llsD+, llsG+	-	-	-	-	-	1	1
	CC6	ST6	I	lntA+, vip–, inlJ–, inlF–, llsA+, llsB+, llsD+, llsG+, llsH+, llsX+, llsY+	-	-	-	-	-	1	1
Total					12	28	9	21	36	19	125

## Data Availability

All the raw sequence reads generated were submitted to the European Nucleotide Archive (http://www.ebi.ac.uk/ena/, accessed on 30 August 2021) under study accession number PRJEB47087.

## Supplementary Materials

The following materials are available online at https://www.mdpi.com/article/10.3390/vetsci8090195/s1, Table S1: Information about the cattle abortion cases, Table S2: Virulence factors of *L. monocytogenes*, Figure S1: the ST genotype of each isolate in the minimum spanning tree.

## References

[1] Schoder D., Melzner D., Schmalwieser A., Zangana A., Winter P., Wagner M. (2011). Important vectors for *Listeria monocytogenes* transmission at farm dairies manufacturing fresh sheep and goat cheese from raw milk. J. Food Prot..

[2] Vazquez-Boland J.A., Kuhn M., Berche P., Chakraborty T., Dominguez-Bernal G., Goebel W., Gonzalez-Zorn B., Wehland J., Kreft J. (2001). Listeria Pathogenesis and Molecular Virulence Determinants. Clin. Microbiol. Rev..

[3] Roberts A.J., Wiedmann M. (2003). Pathogen, host and environmental factors contributing to the pathogenesis of listeriosis. Cell. Mol. Life Sci..

[4] Nightingale K.K., Schukken Y.H., Nightingale C.R., Fortes E.D., Ho A.J., Her Z., Grohn Y.T., McDonough P.L., Wiedmann M. (2004). Ecology and transmission of *Listeria monocytogenes* infecting ruminants and in the farm environment. Appl. Environ. Microbiol..

[5] Dhama K., Karthik K., Tiwari R., Shabbir M.Z., Barbuddhe S., Malik S.V.S., Singh R.K. (2015). Listeriosis in animals, its public health significance (food-borne zoonosis) and advances in diagnosis and control: A comprehensive review. Vet. Q..

[6] Erdogan H.M., Cripps P.J., Morgan K.L., Cetinkaya B., Green L.E. (2001). Prevalence, incidence, signs and treatment of clinical listeriosis in dairy cattle in England. Vet. Rec..

[7] Siegman-Igra Y., Levin R., Weinberger M., Golan Y., Schwartz D., Samra Z., Konigsberger H., Yinnon A., Rahav G., Keller N. (2002). *Listeria monocytogenes* infection in Israel and review of cases worldwide. Emerg. Infect. Dis..

[8] Evans K., Smith M., McDonough P., Wiedmann M. (2004). Eye infections due to *Listeria monocytogenes* in three cows and one horse. J. Vet. Diagnostic Investig..

[9] Drevets D.A., Bronze M.S. (2008). *Listeria monocytogenes*: Epidemiology, human disease, and mechanisms of brain invasion. FEMS Immunol. Med. Microbiol..

[10] Barkallah M., Gharbi Y., Hassena A.B., Slima A.B., Mallek Z., Gautier M., Greub G., Gdoura R., Fendri I. (2014). Survey of infectious etiologies of bovine abortion during mid- to late gestation in dairy herds. PLoS ONE.

[11] Latorre A.A., Van Kessel J.S., Karns J.S., Zurakowski M.J., Pradhan A.K., Boor K.J., Jayarao B.M., Houser B.A., Daugherty C.S., Schukken Y.H. (2010). Biofilm in milking equipment on a dairy farm as a potential source of bulk tank milk contamination with *Listeria monocytogenes*. J. Dairy Sci..

[12] Bandelj P., Jamnikar-Ciglenecki U., Ocepek M., Blagus R., Vengust M. (2018). Risk factors associated with fecal shedding of *Listeria monocytogenes* by dairy cows and calves. J. Vet. Intern. Med..

[13] Rodriguez C., Taminiau B., García-Fuentes E., Daube G., Korsak N. (2021). *Listeria monocytogenes* dissemination in farming and primary production: Sources, shedding and control measures. Food Control.

[14] García J.A., Micheloud J.F., Campero C.M., Morrell E.L., Odriozola E.R., Moreira A.R. (2016). Enteric listeriosis in grazing steers supplemented with spoiled silage. J. Vet. Diagnostic Investig..

[15] Steckler A.J., Cardenas-Alvarez M.X., Townsend Ramsett M.K., Dyer N., Bergholz T.M. (2018). Genetic characterization of *Listeria monocytogenes* from ruminant listeriosis from different geographical regions in the U.S. Vet. Microbiol..

[16] Maury M.M., Tsai Y., Charlier C., Touchon M., Chenal-Francisque V., Leclercq A., Criscuolo A., Gaultier C., Roussel S., Brisabois A. (2016). Uncovering *Listeria monocytogenes* hypervirulence by harnessing its biodiversity. Nat. Genet..

[17] Orsi R.H., den Bakker H.C., Wiedmann M. (2011). *Listeria monocytogenes* lineages: Genomics, evolution, ecology, and phenotypic characteristics. Int. J. Med. Microbiol..

[18] Dreyer M., Aguilar-Bultet L., Rupp S., Guldimann C., Stephan R., Schock A., Otter A., Schüpbach G., Brisse S., Lecuit M. (2016). *Listeria monocytogenes* sequence type 1 is predominant in ruminant rhombencephalitis. Sci. Rep..

[19] Hurtado A., Ocejo M., Oporto B. (2017). *Salmonella* spp. and *Listeria monocytogenes* shedding in domestic ruminants and characterization of potentially pathogenic strains. Vet. Microbiol..

[20] Papić B., Pate M., Félix B., Kušar D. (2019). Genetic diversity of *Listeria monocytogenes* strains in ruminant abortion and rhombencephalitis cases in comparison with the natural environment. BMC Microbiol..

[21] Brown E., Dessai U., Mcgarry S., Gerner-Smidt P. (2019). Use of Whole-Genome Sequencing for Food Safety and Public Health in the United States. Foodborne Pathog. Dis..

[22] Painset A., Björkman J.T., Kiil K., Guillier L., Mariet J.F., Felix B., Amar C., Rotariu O., Roussel S., Perez-Reche F. (2019). Liseq–Whole-genome sequencing of a cross-sectional survey of *Listeria monocytogenes* in ready-to-eat foods and human clinical cases in Europe. Microb. Genom..

[23] Schjørring S., Gillesberg Lassen S., Jensen T., Moura A., Kjeldgaard J.S., Müller L., Thielke S., Leclercq A., Maury M.M., Tourdjman M. (2017). Cross-border outbreak of listeriosis caused by cold-smoked salmon, revealed by integrated surveillance and whole genome sequencing (WGS), Denmark and France, 2015 to 2017. Eurosurveillance.

[24] Doumith M., Buchrieser C., Glaser P., Jacquet C., Martin P. (2004). Differentiation of the major *Listeria monocytogenes* serovars by multiplex PCR. J. Clin. Microbiol..

[25] Bolger A.M., Lohse M., Usadel B. (2014). Trimmomatic: A flexible trimmer for Illumina sequence data. Bioinformatics.

[26] Prjibelski A.D., Puglia G.D., Antipov D., Bushmanova E., Giordano D., Mikheenko A., Vitale D., Lapidus A. (2020). Extending rnaSPAdes functionality for hybrid transcriptome assembly. BMC Bioinform..

[27] Mikheenko A., Prjibelski A., Saveliev V., Antipov D., Gurevich A. (2018). Versatile genome assembly evaluation with QUAST-LG. Bioinformatics.

[28] Wood D.E., Lu J., Langmead B. (2019). Improved metagenomic analysis with Kraken 2. Genome Biol..

[29] Jünemann S., Sedlazeck F.J., Prior K., Albersmeier A., John U., Kalinowski J., Mellmann A., Goesmann A., Von Haeseler A., Stoye J. (2013). Updating benchtop sequencing performance comparison. Nat. Biotechnol..

[30] Ruppitsch W., Pietzka A., Prior K., Bletz S., Fernandez H.L., Allerberger F., Harmsen D., Mellmann A. (2015). Defining and evaluating a core genome multilocus sequence typing scheme for whole-genome sequence-based typing of *Listeria monocytogenes*. J. Clin. Microbiol..

[31] Zhou Z., Alikhan N.F., Sergeant M.J., Luhmann N., Vaz C., Francisco A.P., Carriço J.A., Achtman M. (2018). Grapetree: Visualization of core genomic relationships among 100,000 bacterial pathogens. Genome Res..

[32] Buchfink B., Reuter K., Drost H.G. (2021). Sensitive protein alignments at tree-of-life scale using DIAMOND. Nat. Methods.

[33] Liu B., Zheng D., Jin Q., Chen L., Yang J. (2019). VFDB 2019: A comparative pathogenomic platform with an interactive web interface. Nucleic Acids Res..

[34] Whitman K.J., Bono J.L., Clawson M.L., Loy J.D., Bosilevac J.M., Arthur T.M., Ondrak J.D. (2020). Genomic-based identification of environmental and clinical *Listeria monocytogenes* strains associated with an abortion outbreak in beef heifers. BMC Vet. Res..

[35] Wolf-Jäckel G.A., Hansen M.S., Larsen G., Holm E., Agerholm J.S., Jensen T.K. (2020). Diagnostic studies of abortion in Danish cattle 2015–2017. Acta Vet. Scand..

[36] Nightingale K.K., Fortes E.D., Ho A.J., Schukken Y.H., Grohn Y.T., Wiedmann M. (2005). Evaluation of farm management practices as risk factors for clinical listeriosis and fecal shedding of *Listeria monocytogenes* in ruminants. J. Am. Vet. Med. Assoc..

[37] Castro H., Jaakkonen A., Hakkinen M., Korkeala H., Lindström M. (2018). Occurrence, persistence, and contamination routes of *Listeria monocytogenes* genotypes on three Finnish dairy cattle farms: A longitudinal study. Appl. Environ. Microbiol..

[38] Palacios-Gorba C., Moura A., Gomis J., Leclercq A., Gómez-Martín Á., Bracq-Dieye H., Mocé M.L., Tessaud-Rita N., Jiménez-Trigos E., Vales G. (2021). Ruminant-associated *Listeria monocytogenes* isolates belong preferentially to dairy-related hypervirulent clones: A longitudinal study in 19 farms. bioRxiv.

[39] Šteingolde Ž., Avsejenko J., Berziņš A. (2014). Overview of *Listeria monocytogenes* caused abortions in cattle in Latvia in 2013. Res. Rural Dev..

[40] Kim S.W., Haendiges J., Keller E.N., Myers R., Kim A., Lombard J.E., Karns J.S., Van Kessel J.A.S., Haley B.J. (2018). Genetic diversity and virulence profiles of *Listeria monocytogenes* recovered from bulk tank milk, milk filters, and milking equipment from dairies in the United States (2002 to 2014). PLoS ONE.

[41] Palacios-Gorba C., Moura A., Leclercq A., Gómez-Martín Á., Gomis J., Jiménez-Trigos E., Mocé M.L., Lecuit M., Quereda J.J. (2021). *Listeria* spp. Isolated from Tonsils of Wild Deer and Boars: Genomic Characterization. Appl. Environ. Microbiol..

[42] Lachtara B., Osek J., Wieczorek K. (2021). Molecular typing of *Listeria monocytogenes* IVb serogroup isolated from food and food production environments in Poland. Pathogens.

[43] Pohl M.A., Wiedmann M., Nightingale K.K. (2006). Associations among *Listeria monocytogenes* genotypes and distinct clinical manifestations of listeriosis in cattle. Am. J. Vet. Res..

[44] Terentjeva M., Šteingolde Ž., Meistere I., Elferts D., Avsejenko J., Streikiša M., Gradovska S., Alksne L., Ķibilds J., Bērziņš A. (2021). Prevalence, Genetic Diversity and Factors Associated with Distribution of *Listeria monocytogenes* and Other *Listeria* spp. in Cattle Farms in Latvia. Pathogens.

[45] Soni D.K., Singh M., Singh D.V., Dubey S.K. (2014). Virulence and genotypic characterization of *Listeria monocytogenes* isolated from vegetable and soil samples. BMC Microbiol..

[46] Owusu-Kwarteng J., Wuni A., Akabanda F., Jespersen L. (2018). Prevalence and Characteristics of *Listeria monocytogenes* Isolates in Raw Milk, Heated Milk and Nunu, a Spontaneously Fermented Milk Beverage, in Ghana. Beverages.

[47] Prokop A., Gouin E., Villiers V., Nahori M.-A., Vincentelli R., Duval M., Cossart P., Dussurget O. (2017). OrfX, a Nucleomodulin Required for *Listeria monocytogenes* Virulence. MBio.

[48] Osman K.M., Kappell A.D., Fox E.M., Orabi A., Samir A. (2020). Prevalence, pathogenicity, virulence, antibiotic resistance, and phylogenetic analysis of biofilmproducing *Listeria monocytogenes* isolated from different ecological niches in Egypt: Food, humans, animals, and environment. Pathogens.

[49] Liu D. (2006). Identification, subtyping and virulence determination of *Listeria monocytogenes*, an important foodborne pathogen. J. Med. Microbiol..

[50] Tsai Y.H.L., Orsi R.H., Nightingale K.K., Wiedmann M. (2006). *Listeria monocytogenes* internalins are highly diverse and evolved by recombination and positive selection. Infect. Genet. Evol..

[51] Lebreton A., Lakisic G., Job V., Fritsch L., Tham T.N., Camejo A., Matteï P.J., Regnault B., Nahori M.A., Cabanes D. (2011). A bacterial protein targets the BAHD1 chromatin complex to stimulate type III interferon response. Science..

[52] Cabanes D., Sousa S., Cebriá A., Lecuit M., García-Del Portillo F., Cossart P. (2005). Gp96 is a receptor for a novel *Listeria monocytogenes* virulence factor, Vip, a surface protein. EMBO J..

[53] Quereda J.J., Meza-Torres J., Cossart P., Pizarro-Cerdá J. (2017). Listeriolysin S: A bacteriocin from epidemic *Listeria monocytogenes* strains that targets the gut microbiota. Gut Microbes.

