# Supplementary figures and images for: Characterization and Genetic Diversity of Listeria monocytogenes Isolated from Cattle Abortions in Latvia, 2013–2018
Source: Vet Sci. 2021 Sep 14;8(9):195. doi: 10.3390/vetsci8090195 (PMC8473131; doi:10.3390/vetsci8090195)

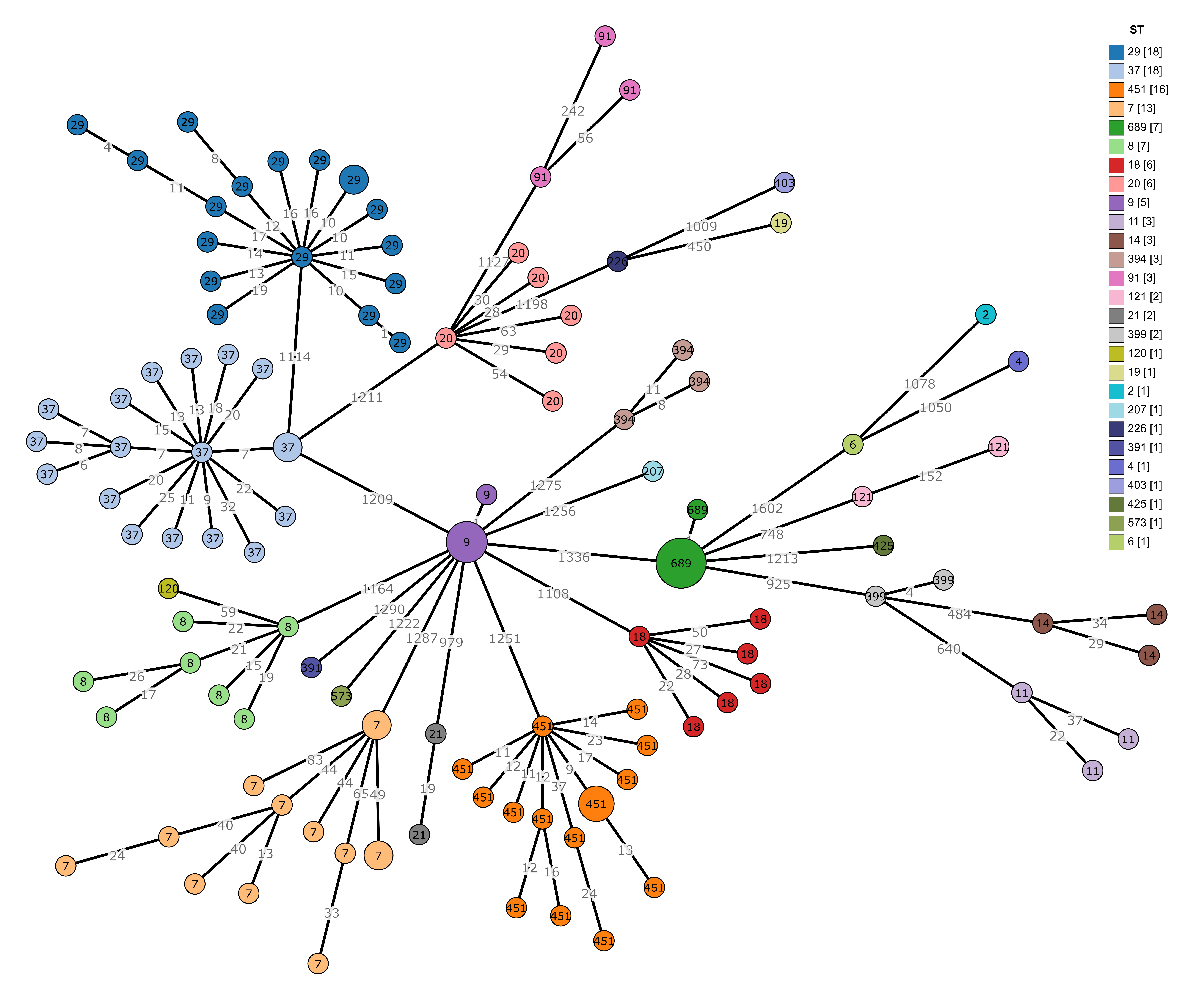

Supplement: Supplementary file 1 [file vetsci-08-00195-s001.zip › Figure S1.png]
